# Influence of Temperature on the Interaction for Resource Utilization between Fall Armyworm, *Spodoptera frugiperda* (Lepidoptera: Noctuidae), and a Community of Lepidopteran Maize Stemborers Larvae

**DOI:** 10.3390/insects11020073

**Published:** 2020-01-21

**Authors:** Bonoukpoè Mawuko Sokame, François Rebaudo, Peter Malusi, Sevgan Subramanian, Dora Chao Kilalo, Gerald Juma, Paul-André Calatayud

**Affiliations:** 1 International Centre of Insect Physiology and Ecology (*icipe*), Nairobi P.O. Box 30772-00100, Kenya; pmalusi@icipe.org (P.M.); ssubramania@icipe.org (S.S.); pcalatayud@icipe.org (P.-A.C.); 2 Department of Plant Science and Crop protection, University of Nairobi, Kangemi, Nairobi P.O. Box 29053-00625, Kenya; ngachalor@gmail.com; 3 CNRS, IRD, UMR Évolution, Génomes, Comportement et Écologie, Université Paris-Saclay, 91198 Gif-sur-Yvette, France; francois.rebaudo@ird.fr; 4 Department of Biochemistry, University of Nairobi, Nairobi P.O. Box 30197-00100, Kenya; gjuma@uonbi.ac.ke

**Keywords:** maize stemborers, fall armyworm, intra- and interspecific, interactions, temperature

## Abstract

Intra- and interspecific interactions within communities of species that utilize the same resources are characterized by competition or facilitation. The noctuid stemborers, *Busseola fusca* and *Sesamia calamistis*, and the crambid stemborer, *Chilo partellus* were the most important pests of maize in sub-Saharan Africa before the recent “invasion” of fall armyworm (FAW), *Spodoptera* *frugiperda,* which currently seriously limits maize yields in Africa. This new pest is interacting with the stemborer community at the larval stage in the use of maize resources. From previous works on the influence of temperature on the larval intra- and interspecific resources utilization within the community of Lepidoptera stemborers involving *B. fusca*, *S. calamistis,* and *C. partellus*, there is a need to update these studies by adding the new pest, *S. frugiperda*, in order to understand the effect of temperature on the larval interactions of all these four species under the context of climate change. The influence of temperature on intra- and interspecific larval interactions was studied using artificial stems kept at different constant temperatures (15 °C, 20 °C, 25 °C, and 30 °C) in an incubator and assessing survival and relative growth rates of each species in single and multi-species experiments. After the inclusion of FAW into the experiments, with regard to relative growth rates, both intra- and interspecific competition was observed among all four species. With regard to survival rates, cannibalism can also explain the intra- and interspecific interactions observed among all four species. Interspecific competition was stronger between the stemborers than between the FAW and the stemborers. Similar to lepidopteran stemborers, temperature affected both survival and relative growth rates of the FAW as well. Regardless of the temperature, *C. partellus* was superior in interspecific interactions shown by higher relative growth and survival rates. The results suggest that the FAW will co-exist with stemborer species along entire temperature gradient, though competition and/or cannibalism with them is weak. In addition, temperature increases caused by climate change is likely to confer an advantage to *C. partellus* over the fall armyworm and the other noctuids.

## 1. Introduction

The noctuids *Busseola fusca* (Fuller) and *Sesamia calamistis* Hampson and the crambid *Chilo partellus* (Swinhoe) were considered as the economically most important pests of maize and sorghum in sub-Saharan Africa [1,2,3,4,5,6] before the recent “invasion” of the fall armyworm (FAW), *Spodoptera frugiperda* (J. E. Smith) (Lepidoptera, Noctuidae) in Africa, which currently limits seriously maize yields [7,8]. In contrast to stemborers, the crop seems to be impacted by FAW at all growth stages from seedling to maturity [7,8]. FAW was first reported from the western region of Kenya in 2017 but by early 2018, it has been confirmed in more than 42 counties throughout the country [9]. This new pest is showing at larval stages frequent interactions with the stemborer community in the use of maize resources [10]. In maize fields, second instar FAW larvae are commonly found in communities of mixed species with stemborer species in the whorl of the plants and, later on, it is frequent to find older FAW larvae in the bored holes and tunnels left by stemborers and to find a FAW larva feeding on corn with stemborer larvae [11], which might lead them to compete with stemborer species, even with *S. calamistis* larvae which is known to feed in a short time on the leaf sheaths and to immediately bore into the stem [4,12].

In 2016, both intra- and interspecific competition was studied at different larval stages and demonstrated for the *B. fusca*-*S. calamistis*-*C. partellus* communities with stronger interspecific competition recorded between the noctuids and the crambid than between the two noctuids [13]. It was also reported in America, interspecific competition between FAW with other species [14,15]. As already stated by Ntiri et al. [13], temperature is a crucial parameter among the abiotic factors driving directly the rate of growth and development, fecundity and mortality, resource utilization, and thus the interspecific interactions. Although unpredictability in rainfall is also a relevant effect of climate change which could be serious driver of caterpillar fitness, Ntiri et al. [16] showed that temperature was the most significant abiotic factor influencing the composition of stemborer communities in Kenya.

From the work done by Ntiri et al. [13] on the influence of temperature on the intra- and interspecific resource utilization within the community of Lepidoptera stemborers involving *B. fusca*, *S. calamistis* and *C. partellus*, there is a need to update this study by adding a new pest, *S. frugiperda*, in order to understand the effect of temperature on the interactions of all these four species under a context of climate change. In this study, competition within individuals of either FAW or of stemborers (*B. fusca*, *S. calamistis* and *C. partellus*) was considered as intraspecific while competition between FAW and a given stemborer larvae was considered as interspecific. The objectives of this study were to investigate the kind of intraspecific and interspecific interactions within and between FAW and stemborers and to evaluate the effect of temperature on these interactions under laboratory conditions.

## 2. Materials and Methods

### 2.1. Plants and Insects

Maize plants of hybrid H513 (Simlaw, Kenya Seed Company, Nairobi, Kenya) were grown in plastic pots of 12 cm in height and 13 cm in diameter in a greenhouse at the Duduville campus of International Centre of Insect Physiology and Ecology (*icipe*), Nairobi, Kenya. Mean temperatures were 29/17 °C (day/night) with an L12:D12 photoperiod. Plants used in experiments were between 4 to 5 weeks old (about 60– 75 cm of size), the earliest stage found to be infested in the field.

Larvae of stemborers (*B. fusca*: Bf, *C. partellus*: Cp, *S. calamistis*: Sc) and FAW were supplied by the Animal Rearing and Containment Unit (ARCU) at *icipe*. These colonies were rejuvenated twice a year with field-collected larvae. Larvae were reared in plastic jars of 16.5 cm length and 9 cm in diameter, filled with 200 mL of the artificial diet described by Onyango and Ochieng’-Odero [17]. For FAW larvae, the diet was modified by adding wheat germ, milk powder, and Suprapen powder and removal of sucrose [18]. To prevent escape of larvae, the jars were tightly closed with tissue paper and perforated lids with a galvanized mesh. The jars were kept in a holding room at 26 ± 1 °C and RH of 62 ± 5%.

### 2.2. Surrogate Stems

Since larvae-infested maize plants kept in the incubator deteriorate after 5–7 days, the method developed by Ntiri et al. [13] using surrogate stems filled with artificial diet was used in this experiment. They consisted of a piece of polymerizing vinyl chloride (PVC) pipe of 30 cm in length and 5 cm in diameter. This was cut into equal halves to allow opening of the stem for recovery of the larvae. The two halves were held together with masking tape and one end was covered with parafilm tightened with masking tape. The pipes were then half filled with artificial diet, leaving about 300 cm^3^ free space. Once the diet became solid in the pipe after 24 h, the masking tape and the aluminum foil covering the pipe were removed from the top to bottom up to three quarters length of the pipe.

### 2.3. Preliminary Experiments: Effects of Diets, Instars, and Rearing Substrates on Survival and Relative Growth Rates of FAW Larvae

The purpose was first to test if the stemborer diet of Onyango and Ochieng’-Odero [16] was also suitable for FAW larvae. Since FAW larvae were shown to be reared on artificial diet from second instar [19], the first instar was reared on maize leaves whereas second and third instar larvae were reared on the stemborer diet (see results). Similar to the protocol of Ntiri et al. [13], ten pipes were prepared with either stemborer or FAW rearing diets and each pipe was infested with either 8 s or third instar FAW larvae using a small camel hair brush and kept in the holding room at 26 ± 1 °C and RH of 62 ± 5%. After 15 days, the survival and relative growth rates of all larvae were evaluated.

Since the fall armyworm is a leaf feeder and not a stemborer, the use of surrogate stems of Ntiri et al. [13] might not be suitable. Thus, three different substrates consisting of entire maize plants, surrogate stems of Ntiri et al. [13] and glass Petri dishes filled with artificial diet were tested under greenhouse conditions. The surrogate stems and Petri dishes partially filled with the artificial diet of Onyango and Ochieng’-Odero [17] (a diet found to be suitable also to fall armyworm larvae [see results]) as well as entire maize plants were each infested with 8 s instar FAW larvae. The plants were individually enclosed in a net (90 cm in height × 33 cm in diameter) equipped with one-way drawstrings mesh cloth bag to restrict the larvae to the plant. The free end of the surrogate stem was plugged with cotton wool after infestation. The stems were placed upright in jar (8 cm in height × 5 cm in diameter) per replicate. Each treatment was replicated ten times. Temperature was recorded with a HOBO Temp/RH data logger (Onset, MA, USA). After 15 days, maize plants were dissected, surrogate stems and glass Petri dishes opened to record the number and the fresh mass of surviving larvae.

### 2.4. Influence of Different Constant Temperatures on Intra- and Interspecific Interactions within the FAW and a Community of Maize Stemborer Species

This experiment involved single species infestation of either fall armyworm or stemborers larvae and multi-species infestation of FAW and a community of stemborer larvae. They were conducted with surrogate stems of Ntiri et al. [13], which were found to be suitable also to FAW larvae (see results). For all species, since second instar FAW larvae are commonly found in communities of mixed species with stemborer species in the whorl of the plants in the fields [11] and that neonates of FAW are not able to feed on artificial diet [19], only second instar larvae were used for all the following infestations on artificial diet [13]. Following the protocol of Ntiri et al. [13], the single-species infestation treatment consisted of 8 larvae. The multi-species infestation involved in four larvae per stem of each species for the FAW+Bf, FAW+Sc, FAW+Cp pairings, three larvae of each stemborers species and two larvae of FAW for the FAW+Sc+Cp, FAW+Bf+Sc, and FAW+Bf+Cp, and two larvae of each species for the FAW+Sc+Bf+Cp.

The surrogate stems, after infestation were plugged with cotton wool and placed in the jars in an upright position. The experiment was carried out in incubators (Sanyo MIR 554, Tokyo, Japan) at four constant temperatures of 15, 20, 25, and 30 °C, with relative humidity 70 ± 10% and a photoperiod of L12:D12. Each treatment was replicated ten times. After 15 days, surrogate stems were opened to record the number and the mass of surviving larvae of each species.

### 2.5. Data Analysis

The outcomes of competition were evaluated through survival rates (proportion of the number of larvae alive after 15 days) and relative growth rates (RGR) as the response variables. The relative growth rate for each species was calculated using the Ojeda-Avila et al. [20] Equation:

$$\mathrm{RGR}=\frac{\mathrm{Mass}\;\mathrm{per}\;\mathrm{surviving}\;\mathrm{larva}-\mathrm{Initial}\;\mathrm{mass}\;\mathrm{per}\;\mathrm{larva}}{\mathrm{Number}\;\mathrm{of}\;\mathrm{days}}$$

For the species communities, RGR was performed as the mean of the RGR of all species in that community. For each treatment, survival rates were analyzed using the generalized linear models (GLM) with binomial error distribution. Significant differences were separated using Tukey’s multiple comparisons tests performed using the R package “lsmeans” [21]. For the comparison performed between treatments from the GLM results, Odd Ratio with a 95% confidence level interval (O.R. [95%CI]) was calculated. From each treatment, the differences between species RGR were analyzed via analysis of variance (ANOVA). The ANOVA was performed by constructing a general linear model with the *lm* function at 5% of level of significance. Significant differences were also separated using Tukey’s multiple comparisons tests performed using the R package “lsmeans” [21] with *p*-value adjustment method = False Discovery Rate (FDR) as addressed by Verhoeven et al. [22]. The RGR data were first tested for normality of their distribution using Shapiro–Wilk test and for homogeneity of variance using Bartlett test. All analyses were performed with R software version 3.5.1 [23]. Within the R environment, other packages such as multcomp [24], MASS [25], Rmisc [26], car [27], FSA [28], Psych [29], and multcompView [30] were used for the analysis.

## 3. Results

### 3.1. Effects of Diets, Larval Instars, and Rearing Substrates on Survival and RGR of FAW Larvae

**Figure 1:**
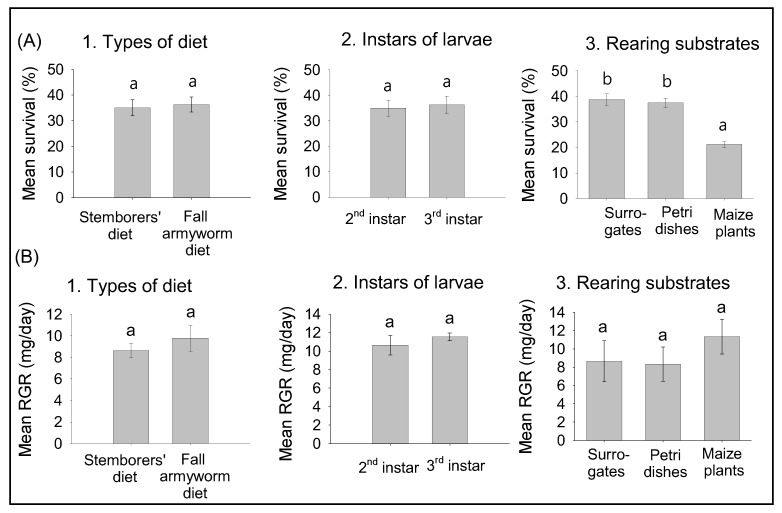
Effects of diet type, larval stage and rearing substrates on the survival (**A**) and relative growth rate (**B**) of fall armyworm larvae. Means (±SE) with different letters are significantly different, determined using linear model (GLM) with binomial error distribution. Significant differences were separated using Tukey’s multiple comparisons tests performed with lsmeans R package [21], following generalized linear model (GLM) with binomial errors distribution. For relative growth rate (RGR), means (±SE) were separated with lsmeans R package [21] with p-value adjustment method False Discovery Rate (FDR) [22] following analysis of variance (ANOVA) after constructing general linear models.

The types of diet tested had no significant effect on survival rates (O.R. = 1.05 (0.85–2.02), *p* = 0.86) and RGRs (*F* = 0.68, *p* = 0.41) (Figure 1(A1,B1)), and no differences in either were found between second and third instars (Survival: O.R. = 1.03 (0.95–1.92), *p* = 0.87; RGRs *F* = 0.62, *p* = 0.43) (Figure 1A2,B2).

While there were no statistical differences between rearing substrates for RGRs (*F* = 2.56, *p* = 0.12), higher larval survival rates were obtained from surrogates and Petri dishes than maize plants (O.R. = 0.66 (0.47–0.93), *p* = 0.01) (Figure 1A3,B3). Therefore, stemborer’s diet and surrogate stems were used for the subsequent experiments on intra- and interspecific interactions using second instar FAW larvae

### 3.2. Influence of Different Constant Temperatures on Intra- and Interspecific Interactions

#### 3.2.1. The Effect of Temperature on Survival and RGR of FAW and Stemborer Larvae

**Figure 2:**
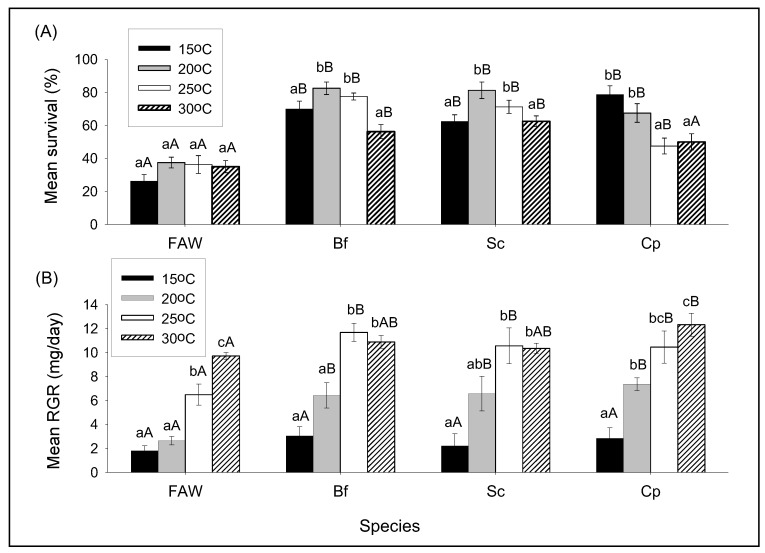
Comparison of survival (**A**) and relative growth rate (**B**) of *S. frugiperda* (FAW), *B. fusca* (Bf), *S. calamistis* (Sc), and *C. partellus* (Cp) in single-species combinations at different constant temperatures. Means (±SE) with different letters are significantly different, determined using Tukey’s multiple comparisons tests performed with lsmeans R package [21], following generalized linear model (GLM) with binomial error distribution for survival or False Discovery Rate (FDR) [22] with lsmeans R package [21], following analysis of variance (ANOVA) after constructing general linear models for relative growth rates. Small letters were used to compare means between temperatures for each species and capital letters to compare means between species for each temperature.

There was no statistical difference in survival rates of FAW larvae between temperatures but there was higher survival of *B. fusca* and *S. calamistis* at both 20 and 25 °C compared to 15 and 30 °C, while *C. partellus* had higher survival rate at both 15 and 20 °C than 25 and 30 °C (Figure 2A, Table 1). Between species at each temperature, the survival rate of FAW larvae was significantly lower than those of each stemborer species larvae except for *C. partellus* at 30 °C (Figure 2A, Table 1).

FAW had similar RGR between 15 and 20 °C where after it increased with increasing temperature (Figure 2B, Table 2). For each stemborer species, RGRs increased significantly with increasing temperature to become similar between 25 and 30 °C (Figure 2B, Table 2). Between species at 15 °C, there were no significant differences in RGRs. When significant at other temperatures, RGRs were lower for FAW than those of stemborers (Figure 2B, Table 2).

#### 3.2.2. Comparison of Survival and RGR of FAW and Lepidopteran Stemborers in Multi-Species Combinations under Different Constant Temperatures

**Figure 3:**
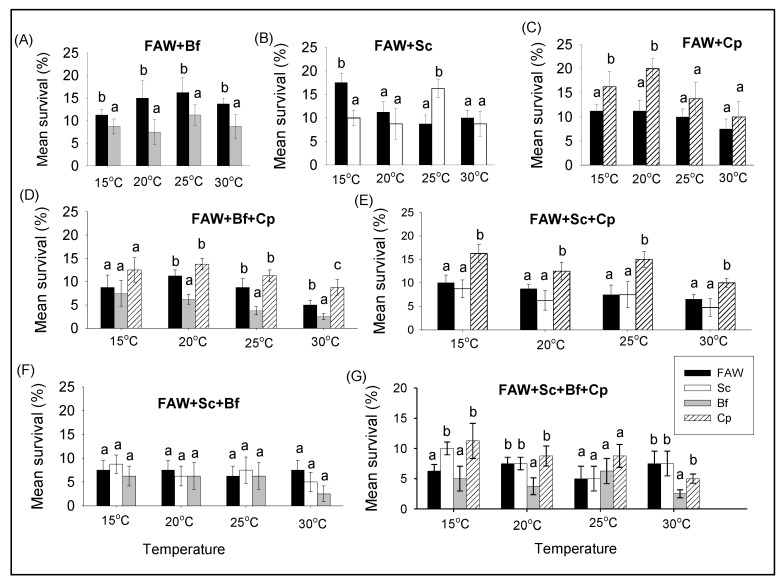
Comparison of survival of *S. frugiperda* (FAW), *B. fusca* (Bf), *S. calamistis* (Sc), and *C. partellus* (Cp) in multi-species combinations at different constant temperatures. Means (±SE) with different letters are significantly different, determined using Tukey’s multiple comparisons tests performed with lsmeans R package [21], following generalized linear model (GLM) with simple binomial procedure. **A** = FAW+Bf, **B** = FAW+Sc, **C** = FAW+Cp, **D** = FAW+Bf+Cp, **E** = FAW+Sc+Cp, **F** = FAW+Sc+Bf, **G** = FAW+Sc+Bf+Cp.

In pairwise combinations, larval survival of FAW was higher than those of *B. fusca* at all temperatures (Figure 3A, Table 3), and higher than those of *S. calamistis* at 15 °C, but it was reverse at 25 °C (Figure 3B, Table 3). In contrast, the survival of *C. partellus* dominated significantly those of FAW at 15 and 20 °C (Figure 3C, Table 3). In the three species combinations, *B. fusca* exhibited the lowest survival rates in the FAW, *B. fusca*, and *C. partellus* combination at 20, 25 and 30 °C (Figure 3D, Table 3) whereas *C. partellus* had the highest survival rate in the FAW, *S. calamistis,* and *C. partellus* combination at all temperatures (Figure 3E, Table 3), while at all temperatures there was no significant difference between species when FAW was in combination with only noctuids (i.e., FAW+Sc+Bf) (Figure 3F, Table 3). In the four species combination, *S. calamistis* and *C. partellus* had higher survival rates than FAW and *B. fusca* at 15 °C while *B. fusca* had the lowest survival rate at 20 °C and 30 °C (Figure 3G, Table 3).

**Figure 4:**
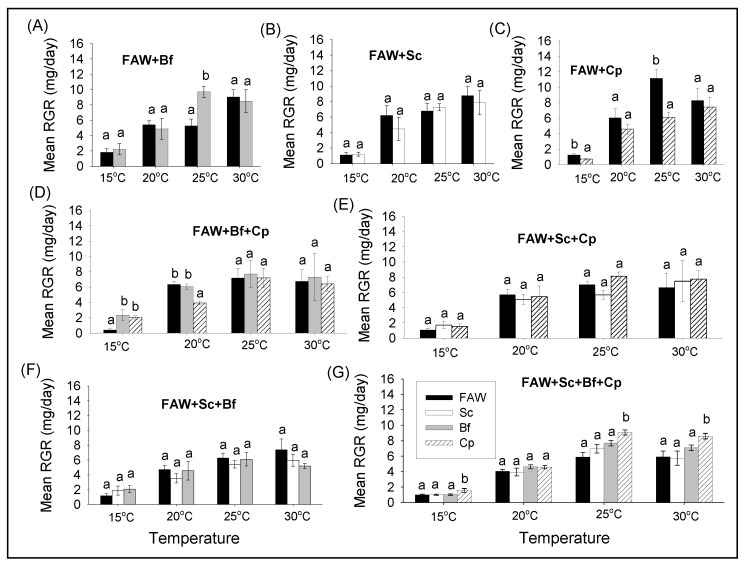
Comparison of relative growth rates of *S. frugiperda* (FAW), *B. fusca* (Bf), *S. calamistis* (Sc), and *C. partellus* (Cp) in multi-species combinations at different constant temperatures. Means (±SE) with different letters are significantly different, determined using False Discovery Rate (FDR) [22] with lsmeans R package [21], following analysis of variance (ANOVA) after constructing general linear models. **A** = FAW+Bf, **B** = FAW+Sc, **C** = FAW+Cp, **D** = FAW+Bf+Cp, **E** = FAW+Sc+Cp, **F** = FAW+Sc+Bf, **G** = FAW+Sc+Bf+Cp.

Except at 25 °C, where *B. fusca* had the highest RGR, no significant difference has been revealed in the FAW and *B. fusca* pairwise combination at all temperatures (Figure 4A, Table 4). Likewise, no significant difference of RGR was found between species in the FAW and *S. calamistis* combination at all temperatures (Figure 4B, Table 4), while in the FAW and *C. partellus* combination, FAW exhibited the highest RGR at 15 and 25 °C (Figure 4C, Table 4). In the FAW, *B. fusca* and *C. partellus* combination, FAW had the lowest RGR at 15 °C while it was the reverse at 20 °C together with *B. fusca* but it was similar at all other temperatures (Figure 4D, Table 4). For the other three species combinations, there were no significant differences in RGRs between species at all temperatures (Figure 4E,F, Table 4). In the combination of the four species, except at 20 °C, *C. partellus* always exhibited the highest RGR (Figure 4G, Table 4).

#### 3.2.3. Comparison of Survival and RGR between Single and Multi-Species Combinations of Fall Armyworm and Lepidopteran Stemborers at Different Constant Temperatures

**Figure 5:**
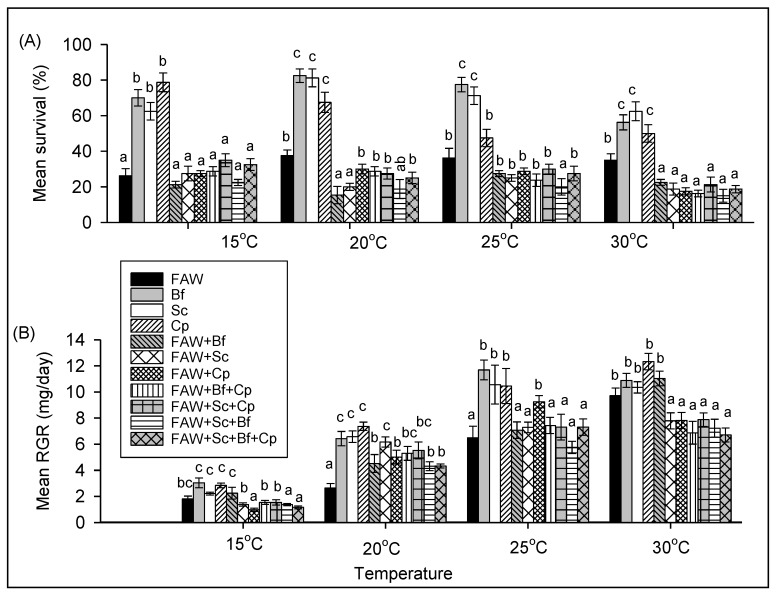
Comparison of survival (**A**) and relative growth rate (**B**) between single-species and multi-species combinations of *S. frugiperda* (FAW), *B. fusca* (Bf), *S. calamistis* (Sc), and *C. partellus* (Cp) under different constant temperatures. Statistical comparisons were only made between single- and corresponding multi-species pairings (see Table 5 and Table 6). Means (±SE) were compared using Tukey’s multiple comparisons tests performed using with lsmeans R package [21], following generalized linear model (GLM) with simple binomial procedure for survival or false discovery rate [22] following analysis of variance (ANOVA) after constructing general linear models for relative growth rates.

When significant, survival and RGR of a single stemborer species were higher than total survival and RGR of the corresponding multi-species communities and higher than that of FAW singly, survival of *B. fusca*, *S. calamistis,* and *C. partellus* singly tended to be higher than that of the total survival of the corresponding multi-species communities. For FAW, it was higher than that of multi-species communities only at temperatures higher than 15 °C (Figure 5A, Table 5). Likewise, RGRs of single-species communities of *B. fusca*, *S. calamistis,* and *C. partellus* tended to be higher than the total RGRs of the corresponding multispecies communities for all temperatures (Figure 5B, Table 6). For FAW, it tended to be higher than that of multi-species communities only at 30 °C, whereas it was lower at 20 °C (Figure 5B, Table 6).

## 4. Discussion

In intraspecific interactions, survival rates tended to decrease with increasing temperatures whereas the RGRs increased. This decrease of survival rates can be due to competition or rather cannibalism. In fact, FAW exhibited the lowest survival rates regardless of temperature. This might be due to the cannibalistic behavior of FAW well reported by several studies under field conditions such as, Sarmento et al. [31], Farias et al. [32], Da Silva [33], and Chapman et al. [34] and under laboratory conditions by De Polanía et al. [35], Goussain et al. [36], Da Silva and Parra [19], Chapman et al. [37,38], and Bentivenha et al. [14]. Cannibalism has been also reported in true stemborers such as the Southwestern corn borer *Diatraea grandiosella* (Dyer) and the European corn borer *Ostrinia nubilalis* (Hubner) (Lepidoptera: Crambidae) [39]. Although it has been less frequently reported in African cereal stemborers, it occurs, for example, among *C. partellus* larvae of the same size at high larval densities [40]. Therefore, the decrease of survival rates in stemborers in our study can be also explained by cannibalism.

Larval survival and RGR of each stemborer tended to decrease under interspecific interactions regardless of the temperature. With regard to RGR, this indicates competitive resource utilization as already reported by Ntiri et al. [13] between stemborer species. With regard to survival, as mentioned before, this could be due to competition or rather cannibalism. By contrast, compared to stemborers, RGRs of FAW across temperatures were less affected by interspecific than intraspecific interactions. The competition–relatedness hypothesis, which states that closely related species are more competitive [41,42] might explain the higher competition among stemborers than between fall armyworm and stemborers as FAW larvae have a different mode of feeding compared to the true stemborers. The lower interspecific competition between FAW and stemborers than within stemborers could be explained by the fact that in contrast to stemborers, which only feed on leaves up to the third larval instar stage (even earlier stage for *S. calamistis*), FAW is a pure foliar feeder; thus, as on live plants, FAW larvae remained on the surface whereas borer larvae penetrated into the diet, where after the direct competition ended between FAW and stemborer larvae. Similarly, Shi et al. [43] reported no evidence of interspecific competition between the rice water weevil, a leaf feeder and rice stemborers at the tillering stage in contrast to the booting or earlier developmental stages of rice.

Regardless of the temperature, the pairwise interactions with FAW reduced survival of *B. fusca* but not of *S. calamistis* and *C. partellus*. This dominance in competitive systems can be the result of competitive inequalities between species [44,45], but also the result of cannibalism inequalities between species. In fact, when FAW was reared together with *B. fusca* and *C. partellus*, it dominated over *C. partellus* in terms of survival but not when it was reared together with *S. calamistis* and *C. partellus*.

In the combinations involving all noctuids (fall armyworm, *B. fusca* and *S. calamistis*) and the crambid (*C. partellus*), the outcomes of interspecific competition were stronger in term of RGRs and skewed asymmetrically towards the crambid, suggesting a higher fitness of the crambid compared to the three noctuids. These results confirm the asymmetry of interspecific competition outcomes in phytophagous insects [42,46,47] and the asymmetrical competition already showed by Ntiri et al. [13] between noctuids, *B. fusca* and *S. calamistis* and the crambid, *C. partellus*. The superiority of *C. partellus* over other stemborer species has been well reported and discussed by Ntiri et al. [13]. In addition, the competitive abilities of each species involved in a competition depend on its temperature tolerance limits for survival and development and thus the outcomes of interspecific competition are greatly affected by temperature [13,15,48,49,50,51,52,53,54,55,56,57]. This was also the case for, the interactions between FAW and the stemborers. Except at 20 °C, *C. partellus* RGR always outcompeted FAW and other stemborers species when reared together; and this dominance over especially FAW was enhanced with increasing temperature. The effect of temperature on competitive abilities of interacting species has been reported between the three stemborer species used in this study [13] who found that the competitive abilities of one of the competing species were enhanced by either low or high temperatures. The coexistence trend in FAW and *S. calamistis* combinations across temperatures might be due to their wider thermal tolerance. In the field, while *C. partellus* and *B. fusca* dominate within a limited thermal tolerance at the high and low temperature extremes, respectively, *S. calamistis* has a wider thermal tolerance by co–occurring with the two species along most of these temperature gradients [58,59,60]. In addition to unpredictability in rainfall, the temperature increase caused by future climate change is likely to confer an advantage on *C. partellus* over FAW and the noctuids in the utilization of maize resources.

## 5. Conclusions

The overall weak competition from second instar between fall armyworm and stemborers indicates that FAW will be able to co-exist with stemborer species along the entire temperature gradient and add to the production constraints of cereal crops. However, with the expanding FAW invasion across agroecologies, studies need to be conducted in the fields along altitudinal gradient to validate the results of the laboratory studies and to predict the trends of population evolution of these species in different agro-climatic zones and how it is likely to evolve with climate change for development of possible management strategies. Additional experiments are needed to understand such interactions at first instar larvae.

## Figures and Tables

**Table 1:** Result of GLM analysis comparing larval survival of each single-species at different constant temperature and at each temperature between species.

Between Temperatures	Statistic Values		Between Species	Statistic Values	
	O.R. (95% CI)	*p*-Value		O.R. (95% CI)	*p*-Value
FAW	1.11 (0.90–1.37)	0.29	15 °C	1.95 (1.57–2.45)	<0.0001
Bf	0.79 (0.63–0.98)	0.04	20 °C	1.51 (1.22–1.89)	0.0001
Sc	0.95 (0.77–1.18)	0.66	25 °C	1.11 (0.91–1.36)	0.02
Cp	0.62 (0.50–0.77)	<0.0001	30 °C	1.22 (1.01–1.50)	0.04

O.R. = odd ratios, CI = confidence interval, FAW = *Spodoptera frugiperda*, Bf = *Busseola fusca*, Sc = *Sesamia calamistis*, Cp = *Chilo partellus.*

**Table 2:** Result of ANOVA comparing relative growth rates of each single-species at different constant temperature and at each temperature between species.

Between Temperatures	Statistic Values		Between Species	Statistic Values	
	*F*	*p*-Value		*F*	*p*-Value
FAW	19.14	<0.0001	15 °C	0.35	0.78
Bf	14.09	<0.0001	20 °C	3.81	0.01
Sc	8.36	0.0002	25 °C	3.66	0.02
Cp	13.01	<0.0001	30 °C	2.80	0.04

FAW = *Spodoptera frugiperda*, Bf = *Busseola fusca*, Sc = *Sesamia calamistis*, Cp = *Chilo partellus*.

**Table 3:** Results of GLM analysis comparing larval survival between fall armyworm vs. stemborer species in multi-species combinations at different constant temperatures.

Temperature	FAW+Bf		FAW+Sc		FAW+Cp		FAW+Bf+Cp	
	O.R. (95% CI)	*p*-Value	O.R. (95% CI)	*p*-Value	O.R. (95% CI)	*p*-Value	O.R. (95% CI)	*p*-Value
15 °C	1.24 (0.82–1.89)	0.03	0.52 (0.19–1.30)	0.01	1.53 (0.61–2.93)	0.03	1.24 (0.73–2.14)	0.42
20 °C	2.17 (1.04–3.54)	0.04	0.75 (0.25–2.13)	0.59	1.97 (0.82–2.95)	0.01	1.14 (0.68–1.92)	0.04
25 °C	0.65 (0.25–1.61)	0.03	2.02 (0.78–5.66)	0.02	1.43 (0.54–3.90)	0.46	1.18 (0.66–2.14)	0.03
30 °C	0.60 (0.21–1.61)	0.04	0.86 (0.28–2.52)	0.78	1.37 (0.45–4.34)	0.57	1.45 (0.72–2.06	0.03
**Temperature**	**FAW+Sc+Cp**		**FAW+Sc+Bf**		**FAW+Sc+Bf+Cp**			
	**O.R. (95% CI)**	***p*-Value**	**O.R. (95% CI)**	***p*-Value**	**O.R. (95% CI)**	***p*-Value**		
15 °C	1.35 (0.83–2.25)	0.02	0.91 (0.50–1.65)	0.76	1.14 (0.79–1.65)	0.02		
20 °C	1.25 (0.73–2.19)	0.03	0.90 (0.47–1.69)	0.75	1.00 (0.67–1.47)	0.03		
25 °C	1.53 (0.90–2.67)	0.01	1.00 (0.53–1.87)	1.00	1.24 (0.82–1.89)	0.30		
30 °C	1.21 (0.65–2.26)	0.03	0.57 (0.25–1.19)	0.15	0.78 (0.50–1.20)	0.02		

O.R. = odd ratios, CI = confidence interval, FAW = *Spodoptera frugiperda*, Bf = *Busseola fusca*, Sc = *Sesamia calamistis*, Cp = *Chilo partellus.*

**Table 4:** Result of ANOVA comparing relative growth rates of fall armyworm vs. stemborer species in multi-species combinations at different constant temperatures.

Temperature	FAW+Bf		FAW+Sc		FAW+Cp		FAW+Bf+Cp	
	*F*	*p*-Value	*F*	P-value	*F*	*p*-Value	*F*	*p*-Value
15 °C	0.22	0.64	0.04	0.83	6.61	0.02	6.67	0.007
20 °C	0.16	0.69	1.78	0.20	1.32	0.26	16.75	<0.0001
25 °C	14.87	0.001	0.27	0.61	14.38	0.002	0.13	0.87
30 °C	0.11	0.74	0.23	0.63	0.16	0.69	0.06	0.93
**Temperature**	**FAW+Sc+Cp**		**FAW+Sc+Bf**		**FAW+Sc+Bf+Cp**			
	***F***	***p*-Value**	***F***	***p*-Value**	***F***	***p*-Value**		
15 °C	0.96	0.39	1.43	0.28	3.70	0.03		
20 °C	0.06	0.94	0.68	0.52	1.04	0.40		
25 °C	0.99	0.39	0.46	0.63	4.86	0.04		
30 °C	0.13	0.87	0.57	0.58	4.08	0.04		

FAW = *Spodoptera frugiperda*, Bf = *Busseola fusca*, Sc = *Sesamia calamistis*, Cp = *Chilo partellus*.

**Table 5:** Results of GLM analysis comparing survival between single-species and multi-species combinations under different constant temperatures.

Comparisons	15 °C		20 °C		25 °C		30 °C	
	O.R. (95% CI)	*p*-Value	O.R. (95% CI)	*p*-Value	O.R. (95% CI)	*p*-Value	O.R. (95% CI)	*p*-Value
FAW vs. FAW+Bf	0.75 (0.36–1.57)	0.45	0.48 (0.23–0.95)	0.04	0.74 (0.38–1.45)	0.39	0.53 (0.36–0.93)	0.008
FAW vs. FAW+Sc	1.06 (0.52–2.15)	0.85	0.41 (0.20–0.83)	0.01	0.58 (0.29–1.15)	0.12	0.42 (0.20–0.87)	0.02
FAW vs. FAW+Cp	1.06 (0.52–2.15)	0.85	0.71 (0.36–1.37)	0.31	0.70 (0.36–1.37)	0.31	0.39 (0.18–0.81)	0.01
FAW vs. FAW+Bf+Cp	1.13 (0.56–2.28	0.72	0.67 (0.34–1.30)	0.24	0.54 (0.27–1.08)	0.08	0.36 (0.16–0.75)	0.007
FAW vs. FAW+Sc+Cp	1.51 (0.77–3.00)	0.23	0.63 (0.32–1.22)	0.17	0.75 (0.38–1.45)	0.40	0.50 (0.24–1.00)	0.05
FAW vs. FAW+Sc+Bf	0.81 (0.39–1.68)	0.58	0.38 (0.18–0.78)	0.009	0.43 (0.21–0.88)	0.02	0.32 (0.14–0.69)	0.004
FAW vs. FAW+Sc+Bf+Cp	1.26 (0.64–2.51)	0.49	0.55 (0.27–1.08)	0.08	0.66 (0.33–1.29)	0.23	0.42 (0.20–0.87)	0.02
Bf vs. FAW+Bf	0.11 (0.05–0.23)	<0.0001	0.06 (0.02–0.13)	<0.0001	0.11 (0.05–0.23)	<0.0001	0.22 (0.11–0.44)	<0.0001
Bf vs. FAW+Bf+Sc	0.12 (0.05–0.24)	<0.0001	0.04 (0.02–0.10)	<0.0001	0.07 (0.03–0.15)	<0.0001	0.13 (0.06–0.28)	<0.0001
Bf vs. FAW+Bf+Cp	0.17 (0.08–0.33)	<0.0001	0.09 (0.04–0.18)	<0.0001	0.09 (0.04–0.18)	<0.0001	0.15 (0.06–0.30)	<0.0001
Bf vs. FAW+Bf+Sc+Cp	0.20 (0.10–0.39)	<0.0001	0.07 (0.03–0.14)	<0.0001	0.11 (0.05–0.22)	<0.0001	0.17 (0.08–0.35)	<0.0001
Sc vs. FAW+Sc	0.22 (0.11–0.43)	<0.0001	0.05 (0.02–0.12)	<0.0001	0.13 (0.06–0.26)	<0.0001	0.13 (0.06–0.27)	<0.0001
Sc vs. FAW+Bf+Sc	0.17 (0.08–0.34)	<0.0001	0.05 (0.02–0.11)	<0.0001	0.10 (0.04–0.20)	<0.0001	0.10 (0.04–0.22)	<0.0001
Sc vs. FAW+Sc+Cp	0.32 (0.16–0.61)	0.0005	0.08 (0.04–0.18)	<0.0001	0.17 (0.08–0.33)	<0.0001	0.16 (0.07–0.32)	<0.0001
Sc vs. FAW+Bf+Sc+Cp	0.28 (0.14–0.54)	0.0001	0.07 (0.03–0.15)	<0.0001	0.15 (0.07–0.30)	<0.0001	0.13 (0.06–0.27)	<0.0001
Cp vs. FAW+Cp	0.10 (0.04–0.20)	<0.0001	0.20 (0.10–0.39)	<0.0001	0.44 (0.22–0.85)	0.01	0.21 (0.10–0.42)	<0.0001
Cp vs. FAW+Bf+Cp	0.10 (0.05–0.21)	<0.0001	0.20 (0.10–0.39)	<0.0001	0.34 (0.17–0.67)	0.002	0.19 (0.08–0.39)	<0.0001
Cp vs. FAW+Sc+Cp	0.14 (0.07–0.28)	<0.0001	0.18 (0.09–0.35)	<0.0001	0.47 (0.24–0.90)	0.024	0.26 (0.13–0.53)	0.0002
Cp vs. FAW+Bf+Sc+Cp	0.12 (0.06–0.25)	<0.0001	0.16 (0.07–0.31)	<0.0001	0.41 (0.21–0.80)	0.009	0.23 (0.11–0.46)	<0.0001

O.R. = odd ratios, CI = confidence interval, FAW = *Spodoptera frugiperda*, Bf = *Busseola fusca*, Sc = *Sesamia calamistis*, Cp = *Chilo partellus.*

**Table 6:** Results of ANOVA comparing the relative growth rates between single-species and multi-species combinations under different constant temperatures.

Comparisons	15 °C		20 °C		25 °C		30 °C	
	*F*	*p*-Value	*F*	*p*-Value	*F*	*p*-Value	*F*	*p*-Value
FAW vs. FAW+Bf	0.79	0.38	6.34	0.02	0.59	0.45	5.87	0.02
FAW vs. FAW+Sc	0.34	0.56	9.69	0.006	0.09	0.76	12.03	0.002
FAW vs. FAW+Cp	13.25	0.002	14.27	0.001	24.34	0.0001	7.77	0.01
FAW vs. FAW+Bf+Cp	0.30	0.59	17.91	0.0005	0.1.79	0.19	10.68	0.004
FAW vs. FAW+Sc+Cp	1.33	0.30	15.99	0.0009	0.57	0.46	7.14	0.01
FAW vs. FAW+Sc+Bf	6.34	0.02	21.83	0.0002	1.73	0.20	12.57	0.002
FAW vs. FAW+Sc+Bf+Cp	12.13	0.002	19.61	0.0003	1.23	0.28	37.86	<0.0001
Bf vs. FAW+Bf	0.75	0.39	9.98	0.005	22.15	0.0001	0.008	0.92
Bf vs. FAW+Bf+Sc	64.26	<0.0001	14.91	0.001	39.72	<0.0001	12.5	0.002
Bf vs. FAW+Bf+Cp	67.61	<0.0001	2.89	0.10	18.45	0.0004	21.73	0.0002
Bf vs. FAW+Bf+Sc+Cp	80.87	<0.0001	23.43	0.0001	19.74	0.0003	42.83	<0.0001
Sc vs. FAW+Sc	22.05	0.0001	0.12	0.92	4.51	0.04	16.44	0.0008
Sc vs. FAW+Bf+Sc	34.72	<0.0001	17.68	0.0006	37.16	<0.0001	9.00	0.008
Sc vs. FAW+Sc+Cp	8.65	0.008	3.61	0.07	19.4	0.0003	5.32	0.03
Sc vs. FAW+Bf+Sc+Cp	43.41	<0.0001	28.12	<0.0001	13.39	0.001	31.78	<0.0001
Cp vs. FAW+Cp	108.1	<0.0001	13.95	0.001	3.40	0.08	11.43	0.003
Cp vs. FAW+Bf+Cp	8.18	0.01	10.72	0.004	16.01	0.0009	16.82	0.0008
Cp vs. FAW+Sc+Cp	29.99	<0.0001	6.48	0.02	8.42	0.009	9.15	0.008
Cp vs. FAW+Bf+Sc+Cp	95.69	<0.0001	61.09	<0.0001	16.4	0.0007	25.93	<0.0001

FAW = *Spodoptera frugiperda*, Bf = *Busseola fusca*, Sc = *Sesamia calamistis*, Cp = *Chilo partellus*.
